# Analysis of the impact of the armed conflict in Ukraine on the population of Romania

**DOI:** 10.3389/fpubh.2022.964576

**Published:** 2022-07-29

**Authors:** Flavius Cristian Mărcău, Cătălin Peptan, Horaţiu Tiberiu Gorun, Vlad Dumitru Băleanu, Victor Gheorman

**Affiliations:** ^1^Faculty of Education, Law and Public Administration, “Constantin Brâncuşi” University of Târgu Jiu, Târgu Jiu, Romania; ^2^“Constantin Brâncuşi” University of Târgu Jiu, Târgu Jiu, Romania; ^3^Faculty of Medical and Behavioural Sciences, “Constantin Brâncuşi” University of Târgu Jiu, Târgu Jiu, Romania; ^4^Faculty of Medicine, University of Medicine and Pharmacy of Craiova, Craiova, Romania

**Keywords:** quality of life, war, Ukraine, Russia, fear, Romania

## Abstract

**Purpose:**

The study aims to highlight the behavior of people in a state in the vicinity of a military conflict zone. Specifically, it highlights the psychological behavior of Romanian citizens after the invasion of Ukraine by the Russian Federation. It was considered appropriate to carry out this study, given the novelty of such a situation, since, after the end of the Second World War, Europe has no longer faced major problems of insecurity caused by armed conflicts of this magnitude.

**Methods:**

The study was based on the questionnaire applied to a number of 1,193 people with permanent residence in Romania and a minimum age of 18 years. The data were collected in the beginning phase of the invasion of Ukraine by the troops of the Russian Federation, i.e. between March 1–17, 2022. The aim was to obtain information that would allow the observation of re-spondents' opinions on the conflict in Ukraine and its potential escalation, and on the other hand, to allow the assessment of quality of life, using the WHQOL-BREEF measurement instrument.

**Results:**

Based on the results of the study, the highest average satisfaction among the four domains of WHOQOL-BREF is represented by the “Psychological” domain, of the category of people with the lowest fear about a potential future war between Romania and the Russian Federation (83.62 ± 17.48). On the contrary, the lowest average is represented by the “Environment” domain, for the category of persons who do not feel protected by the fact that Romania is a NATO member state (61.77 ± 20.96).

**Conclusions:**

The results of the study show that the indices of the quality of life of the people in Romania, as a state in the proximity of a military conflict with the potential to escalate, are negatively influenced by the fears of people who believe that the war in Ukraine will escalate into a regional or global conflict, or that the Russian Federation is going to use its nuclear arsenal against Ukraine or another NATO member state.

## Introduction

The Union of Soviet Socialist Republics (USSR) de jure collapsed in 1991, although it had been a state of collapse since 1989, as a result of anti-totalitarian demonstrations in Central and Eastern Europe and the implosion of the communist regime (1). The new independent states that emerged from the collapse of the USSR remained largely within the sphere of influence of the Russian Federation, the de facto successor of the former USSR. Thus, shortly afterwards, various disputes began between the post-communist states: Armenia and Azerbaijan in 1991–1994 and 2020, following the Nagorno-Karabakh region dispute; Tajikistan between 1992 and 1997; Abkhazia and South Ossetia between 1991 and 1993 and 1998; Transnistria between 1990 and 1992. It should be noted that the Russian Federation has been indirectly involved, through proxy states, in most of the conflicts that have arisen near its borders (2).

In 2014, against the background of internal dissensions in Ukraine caused by the expression of attachment to the values of the West, it was possible for an epicenter of insecurity to emerge, which was followed by the illegal annexation of the Crimean Peninsula by the Russian Federation (3). Such an action was condemned by the leaders of Western states, and economic sanctions were also imposed on the Russian Federation (4). However, many Russian intellectuals believe that Ukraine has no right to exist as an independent state, arguing that it is an artificial construction, and that Ukrainians and Russians are the same people and share the same culture (5).

The annexation of the Crimean Peninsula by the Russian Federation was only the beginning of a long period of tension between pro-Russians and pro-Westerners in the Crimean, Donetsk and Luhansk areas.

On February 24, 2022, the Russian Federation invaded Ukraine, justifying its actions by the so-called desire to denazify (6) and protect Russian nationals on the territory of Ukraine. The actions of the Russian Federation have been severely criticized and sanctioned by the international community, bringing down a new Iron Curtain over Europe, stretching from Norway, from the Barents Sea to Turkey, to the Adriatic Sea, behind which, so far, the states of Belarus and the Russian Federation are located (7). For the first time since the end of Second World War, a military conflict has arisen in Europe which, if it escalates, can bring together the constituent elements of a potential regional or global conflict.

The political implications of the so-called “special military operations” initiated by the Russian Federation are particularly important, given the impact it has on the whole world, so that the regional and global geopolitical and security architecture may undergo profound changes.

In the context of the crisis in Ukraine and the aggressive rhetoric of the Russian Federation with expansionist overtones, some neighboring states - which felt that their national security interests or their political or economic interests were affected - such as Sweden and Finland, or Ukraine, Georgia and the Republic of Moldova, have initiated steps to join NATO or the European Union - which may substantially alter the geopolitical architecture of the European continent, with implications also at global level.

On the other hand, the adoption and application of political and economic sanctions against the Russian Federation by some countries in the democratic world has given new meaning to relations between the BRICS countries (Russian Federation, China, India, Brazil and South Africa) - given their demographic potential and their economic and geopolitical importance - and the idea of a 'new world order' is being put forward in international political circles, in which the BRICS countries would counterbalance the influence of the United States and their NATO partners.

A novelty on the international relations scene is that this armed conflict has succeeded in a very short time in uniting many of the states of the world, around a common goal - the desire for world peace - in a way that no one has been able to do so in the last half century.

It must be acknowledged, however, that the resurgence of armed conflict in Europe has led to many changes in the psychological state of the population. The fear of war has seriously affected the behavior of people living in the states in the immediate vicinity of Ukraine, creating a series of potentially apocalyptic scenarios in the collective mind. The emergence of the crisis in Ukraine at a temporary moment characterized by the global COVID-19 pandemic has superimposed a military security threat on top of a medical security threat, the cumulative effects of which can be seen in increased reactive symptoms of depression-anxiety among the affected population. The population was overwhelmed by anxiety, and in some cases the background anxiety reached the intensity of real panic attacks. Anxiety is described by mental health specialists as fear without purpose (8). Anxiety is usually anticipatory in nature. People who suffer from anxiety experience an intense and often prolonged fear of possible future events (9). In other words, the feeling of uncertainty and the inability to anticipate the short and medium-term perspective, generates a major discomfort that visibly alters the quality of life (10). Clearly, it is very difficult to distinguish between the two types of threat - military and pandemic, which have totally different causalities, manifestations and evolutions - on the behavior of the affected population. By the way of questionnaire design, the present study focuses on highlighting the respondents' quality of life assessment indices on the four major domains (“physical,” “environmental,” “psychological,” and “social”), determined only by the influence of the armed conflict in Ukraine, while the cumulative influences of the pandemic crisis could be a future direction of study.

**Figure 1:**
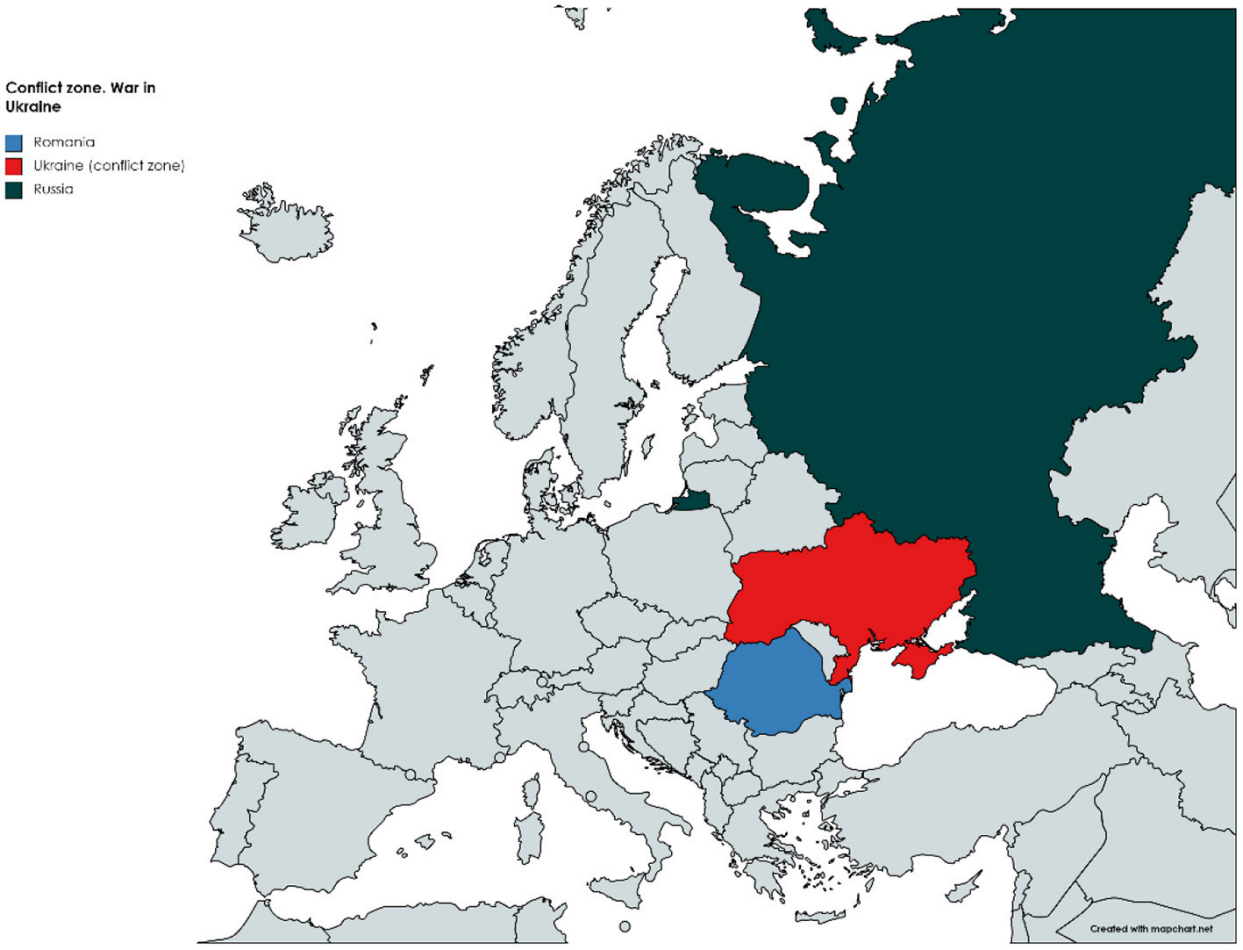
Conflict zone. War in Ukraine. Source: This map was created on www.mapchart.net/.

The armed conflict in Ukraine (Figure 1) is a novelty for the adult population in Romania due to its gravity, complexity and the possibility of escalation, so as to directly affect Romania. From a psychological point of view, the new situation creates a period of uncertainty and fear among the population, so it is necessary to assess their perceptions of the conflict in Ukraine and to measure their quality of life, starting with the first days of the conflict.

The hypothesis of this research is based on the fact that the citizens of Romania, as a state in the immediate vicinity of the war, developed feelings of fear, apprehension and worry about the possible escalation of the conflict in a regional or global one, so that their quality of life indices suffered from the moment the Russian Federation decided to invade Ukraine.

The objectives of the research are to validate/invalidate the research hypothesis, through the answers provided to a set of predetermined questions (see Table 1), based on the processing of data obtained through the questionnaire applied. In this way it will be possible to conclude on the existence of certain correlations between the selected variables in order to complete our study.

**Table 1:** Required data and research questions.

**Required data**	**Questions**
The possibility of war between Romania and the Russian Federation in the near future	Have people who believe in a potential war between Romania and the Russian Federation in the near future developed such a fear as a result of Romania's NATO membership and the presence of foreign troops on Romanian territory?
The belief that the Russian Federation has planned to invade Romania or another NATO member state	Do people who believe in the imminent war of between the Russian Federation and Romania have lower indices of the quality of life compared to people who do not believe that?
The belief that the President of the Russian Federation, Vladimir Putin, will start a nuclear war against NATO member states (including Romania)	
The degree of the population's perception of their personal security, as a result of Romania being a NATO member state	Do people who have a high degree of trust in NATO have better indices of the quality of life than people who have a low degree of trust?
The degree of the population's perception of Romania's security, as a result of the presence of NATO's military capabilities on its territory	
The degree of the population's perception of NATO member states' intervention in case of an attack by the Russian Federation on Romania	

## Research methods

### Participants

The study was conducted between 1 and17 March, 2022, starting on the fifth day of the invasion of the Russian Federation in Ukraine, and consisted of an online questionnaire (social networks and websites) administered to adults in Romania. The receipt of responses is shown in Table 2.

**Table 2:** The timing of this survey according with Russia-Ukraine war days.

**Days of survey**	**Days of war**	**Numbers of responses**
1	5	132
2	6	144
3	7	115
4	8	98
5	9	75
6	10	72
7	11	51
8	12	57
9	13	81
10	14	60
11	15	27
12	16	33
13	17	46
14	18	54
15	19	33
16	20	67
17	21	48
		Total = 1,193

Any person, with a permanent residence in Romania and aged 18 years or older could participate in completing the questionnaire.

Participation in the research was voluntary, anonymous and unpaid. No data were collected on the identifiers of the respondents.

### Procedure

A questionnaire developed on the Google Forms platform was applied to the study participants, and was distributed via a web link. The questionnaire could only be completed by those who ticked “Yes” to the question concerning the permanent residence in Romania and the minimum age of 18 years.

### Measurements

The questionnaire consisted of 46 questions and was structured in two parts: (1) Acquiring socio-demographic and opinion data on the war in Ukraine and the degree of insecurity felt by respondents feel as a result of Romania being located in the immediate vicinity of the conflict and (2) measuring the quality of life of participants.

The questions in the questionnaire were written in Romanian and aimed to determine the participants' opinions on the armed conflict in the vicinity of Romania.

Thus, the information obtained in the first part of the questionnaire allowed the comparison of the quality of life of the participants in order to validate/invalidate the research hypotheses, by providing answers to the questions in Table 1.

In order to determine the quality of life of the participants, the WHOQOL-BREEF measurement instrument, consisting of 26 questions, was applied, given that the WHOQOL-100 may be too broad to be used in large- studies. The quality of life, according to the WHO, is a subjective, psychological state, which implies that a self-assessment questionnaire is the most appropriate for measuring it (11). Regarding the step of checking and cleaning the data and calculating the scores of the major domains, the WHOQOL User Manual was used (12).

### Statistical analysis of data

In order to process the data obtained through the questionnaire, Excel programs, part of the Microsoft Office Professional Plus 2021, and IBM SPSS Statistics 26 were used. These were installed on a computer with the Windows 11 Professional operating system.

The data collected through the questionnaire were centralized in an Excel file and then visualized, extracted and statistically analyzed.

The variables used for the analysis concerned the participants' opinion on: (1) the possible invasion of Romania by the Russian Federation; (2) the possible invasion of a NATO member state by the Russian Federation; (3) the possibility of the outbreak of a nuclear war as a result of the decision of the President of the Russian Federation; (4) the participants' perception of Romania's security in terms of NATO membership.

The data extracted from the questionnaire were statistically analyzed by applying descriptive statistics in order to determine the distribution frequencies, percentages, average scores and standard deviation. In order to determine the degree of correlation, the average scores of the quality of life, among the variables, the Pearson test was applied, and the Kendell and Spearman tests were applied to determine the correlation between the variables extracted from the first part of the questionnaire.

T-tests and one-way ANOVA tests were applied to compare the mean differences. Statistical significance was set as a *P*-value < 0.05.

## Results

The questionnaire was applied to a number of 1,193 people, their socio-demographic data being presented in Table 3.

**Table 3:** Socio-demographic data of the participants.

**Age**	% of the Romanian population*	**Sex**				**Environment of residence**		**Educational level**				
		**Female**		**Male**		**Urban**	**Rural**	**Secondary education**	**High school**	**Faculty**	**Masters**	**PhD**
		***N***	**%**	***N***	**%**							
18–25	10.6%*	253	21.2%	209	17.5%	243	219	1	280	156	25	-
26–30		80	6.7%	71	5.9%	109	42	–	40	67	42	2
31–35		62	5.2%	71	5.9%	95	38	–	23	56	51	3
36–40	34.5%*	63	5.2%	64	5.3%	100	27	1	31	58	33	4
41–45		45	3.77%	57	4.7%	74	28	–	18	47	27	10
46–50		35	2.93%	38	3.1%	57	16	1	16	35	12	9
51–55		32	2.68%	31	2.6%	50	13	–	22	19	13	9
56–60	19.9%*	12	1.01%	15	1.2%	25	2	–	5	8	10	4
61–65		11	0.92%	21	1.7%	27	5	1	9	13	4	5
66+	19.3%*	8	0.67%	15	1.2%	17	6	2	6	11	2	2

^*^2021 Romania population by age group according to Eurostat (https://ec.europa.eu/eurostat).

### Participants' perception of the war in Ukraine

Regarding the Russian Federation's invasion of Ukraine, 95.4% of respondents are aware of it. The sources of information since the beginning of the conflict have been varied, with 63.8% of respondents considering that they had access to reliable sources, which provided credible information about the events in the neighboring state. However, “fake news” information is present in the public space, especially on social media, and is responsible for distorting the truth by presenting false or truncated information in order to manipulate public opinion about the ongoing armed conflict. Thus, 79.6% of respondents believe that false or misleading information can cause panic among the Romanian population.

Fear of a possible war caused by the invasion of the Russian Federation in Romania is present in 47.1% of respondents, while 38.1% of them believe that Romania or another NATO member state will be invaded by the Federation Russian (see Table 4).

**Table 4:** Participants' opinion on a possible armed attack by the Russian Federation on Romania or another NATO member state.

	**Are you afraid of a possible war between Romania and the Russian Federation in the near future? (Q4)**			**To what extent do you think the Russian Federation is planning to invade Romania or another NATO member state? (Q5)**		
(5) To a very large extent	30.2%			14.8%		
(4) To a large extent	16.8%			17%		
(3) Neutral	23.1%			27.3%		
(2) To a small extent	12.1%			17.9%		
(1) To a very small extent	17.6%			22.8%		
Descriptive statistics	Mean		3.30	Mean		2.83
	Standard error	0.042		Standard error	0.039	
	Standard deviation	1.455		Standard deviation	1.353	
	Variance	2.116		Variance	1.831	
	Kurtosis	−1.246		Kurtosis	−1.129	
	Skewness	−0.284		Skewness	0.116	

Weapons of mass destruction, especially nuclear weapons, create serious fears among the Romanian population, with 30.3% of respondents believing that the Russian Federation will launch a nuclear attack against Ukraine, and 24.5% believing that a nuclear attack on NATO member states is a realistic and possible scenario (see Table 5).

**Table 5:** Participants' opinion on a possible nuclear attack launched by the Russian Federation on Romania or another NATO member state.

	**To what extent do you think the Russian Federation will launch a nuclear attack on Ukraine? (Q6)**			**To what extent do you think the President of the Russian Federation, Vladimir Putin, will start a nuclear war against NATO member states? (Q7)**		
(5) To a very large extent	15.7%			12.5%		
(4) To a large extent	14.5%			11.9%		
(3) Neutral	26.8%			25.5%		
(2) To a small extent	18.3%			20.1%		
(1) To a very small extent	24.8%			29.7%		
Descriptive statistics	Mean		2.79	Mean		2.58
	Standard error		0.040	Standard error		0.039
	Standard deviation		1.377	Standard deviation		1.355
	Variance		1.895	Variance		1.935
	Kurtosis		−1.143	Kurtosis		−1.005
	Skewness		0.184	Skewness		0.387

Romania benefits from the presence of NATO troops on its territory in order to strengthen its eastern flank, as a result of the politico-military commitments. However, 23.3% of respondents do not feel protected by Romania's NATO membership, but 57.8% of respondents believe that the presence of the Alliance's military capabilities on Romanian territory is beneficial to state security (see Table 6).

**Table 6:** The opinion of the participants regarding the state of Romania as a NATO member state.

	**To what extent do you feel protected by the fact that Romania is a NATO member state? (Q8)**			**To what extent do you consider the presence of NATO military capabilities on the territory of our state to be beneficial for Romania's security? (Q9)**		
(5) To a very large extent	24.8%			35.2%		
(4) To a large extent	23.8%			22.6%		
(3) Neutral	27.9%			25.3%		
(2) To a small extent	12.3%			7.9%		
(1) To a very small extent	11%			8.8%		
Descriptive statistics	Mean		3.39	Mean		3.68
	Standard error		0.037	Standard error		0.037
	Standard deviation		1.283	Standard deviation		1.270
	Variance		1.646	Variance		1.613
	Kurtosis		−0.862	Kurtosis	−0.579	
	Skewness		−0.366	Skewness		−0.643

It should be noted that NATO leaders reiterated at the Brussels Summit on 24 March 2022 their firm commitment to the collective defense of the Allies (13). In the unlikely event that the Russian Federation launches an attack on Romania or any other NATO member state, Article 5 of the NATO Charter obliges the Allies to intervene against the aggressor state (14). However, 15.8% of respondents believe that Romania will not receive help from Allies in the event of military aggression by the Russian Federation, and 23.2% believe that the US will not intervene in a potential military conflict between Romania and the Russian Federation (see Table 7).

**Table 7:** The opinion of the participants regarding the NATO response in case of an invasion of Romania by the Russian Federation.

	**Do you think that in case of an attack by the Russian Federation on Romania, the NATO member states will come to our aid? (Q10)**			**To what extent do you think that in the event of an attack by the Russian Federation on Romania, the USA will not intervene in the conflict and leave Romania to fight alone? (Q11)**		
(5) To a very large extent	37.8%			12%		
(4) To a large extent	24.7%			11.1%		
(3) Neutral	22%			25.1%		
(2) To a small extent	8.8%			21.3%		
(1) To a very small extent	7%			30.2%		
Descriptive statistics	Mean		3.77	Mean		2.53
	Standard error		0.036	Standard error		0.039
	Standard deviation		1.236	Standard deviation		1.343
	Variance		1.527	Variance		1.903
	Kurtosis		−0.452	Kurtosis		−0.939
	Skewness		−0.728	Skewness		0.440

The participants believe that 38.3% of the Russian Federation's invasion of Ukraine is an event that has influenced their lives (see Table 8).

**Table 8:** Participants' views on the influence of the Ukrainian conflict on their lives.

	**Does the current military conflict in Ukraine have any influence on your life? (Q16)**		
(5) To a very large extent	20.2%		
(4) To a large extent	18.1%		
(3) Neutral	27.8%		
(2) To a small extent	15%		
(1) To a very small extent	18.6%		
Descriptive statistics	Mean		3.35
	Standard error		0.034
	Standard deviation		1.191
	Variance		1.418
	Kurtosis		−0.684
	Skewness		−0.297

### Quality of life of participants

Participants' quality of life varies depending on the variables being reported to, so in terms of the answer to the question “How do you rate your quality of life during the conflict in Ukraine?”, the median of the answers was around 3.26 ± 0.029 (see Table 9).

**Table 9:** The participants' answer to the question “How do you rate your quality of life during the conflict in Ukraine?”

	***N***	**Minimum**	**Maximum**	**Mean**		**SD**	**Variance**
	**Statistic**	**Statistic**	**Statistic**	**Statistic**	**SE**	**Statistic**	**Statistic**
Q1	1,193	1	5	3.26	0.029	1.004	1.009

The total values, according to the four major domains (Physical, Psychological, Social and Environmental), are between 70.57 ± 18.45 and 78.77 ± 19.12 (see Table 10).

**Table 10:** Descriptive statistical analysis of quality of life, on the whole sample, according to the four major areas.

	***N***	**Minimum**	**Maximum**	**Mean**	**SD**
PHYS	1,193	3.57	100.00	70.8718	18.06358
PSYCH	1,193	0.00	100.00	78.7755	19.12893
SOCIAL	1,193	0.00	100.00	74.0360	22.70409
ENVIR	1,193	3.13	100.00	70.5758	18.45976

### Association between socio-demographic data and WHOQOL-BREEF

The comparative analysis of the four major domains, depending on the socio-demographic data of the participants and the answers given to the specific questions in the first part of the questionnaire are shown in Table 11.

**Table 11:** The association between socio-demographic data, specific questions and WHQOL-BREEF.

		**Physical health**	**Psychological health**	**Social relationship**	**Environmental health**	**Quality of life (QOL)**	**Health satisfaction**
Gender	Male	74.45 (17.47)	83.34 (17.52)	75.84 (21.97)	73.23 (17.67)	3.39 (1.04)	4.02 (0.975)
	Female	69.16 (18.10)	77.07 (19.62)	73.17 (23.00)	69.30 (18.70)	3.20 (0.979)	3.88 (1.00)
		*P* = 0.761	*P* = 0.903	*P* = 0.503	*P* = 0.173	*P* = 0.790	*P* = 0.713
Studies	Middle and high school	71.08 (17.96)	77.92 (19.12)	73.66 (22.62)	70.69 (18.92)	3.23 (1.05)	4.01 (1.00)
	University studies	70.74 (18.13)	79.30 (19.1)	74.26 (22.76)	74.25 (22.76)	3.28 (0.970)	3.88 (0.991)
		*P* = 0.914	*P* = 0.730	*P* = 0.635	*P* = 0.456	*P* = 0.235	*P* = 0.274
Environment of residence	Urban	70.48 (18.11)	77.79 (19.16)	73.73 (22.55)	70.26 (18.07)	3.27 (0.972)	3.89 (0.992)
	Rural	71.64 (17.95)	80.77 (18.93)	74.64 (23.02)	71.20 (19.22)	3.25 (1.068)	4.01 (1.01)
		*P* = 0.887	*P* = 0.721	*P* = 0.349	*P* = 0.159	*P* = 0.839	*P* = 0.118
Age	<30	71.55 (17.99)	76.93 (20.29)	74.22 (22.94)	72.14 (18.30)	3.27 (1.057)	4.09 (0.972)
	>30	70.15 (18.12)	80.71 (17.62)	73.83 (22.47)	68.92 (18.49)	3.26 (0.946)	3.75 (1.001)
		*P* = 0.072	*P* = 0.977	*P* = 0.829	*P* = 0.644	*P* = 0.263	*P* = 0.520
Q4^*a*^	1–2	76.48 (17.03)	83.62 (17.48)	76.61 (21.95)	74.37 (16.90)	3.47 (1.08)	4.12 (0.940)
	4–5	67.23 (18.33)	75.22 (19.80)	72.15 (23.80)	67.73 (19.35)	3.14 (1.00)	3.82 (1.04)
		*P* =0.114	*P* = 0.463	*P* = 0.409	*P* = 0.665	*P* = 0.394	*P* = 0.674
Q5^*b*^	1–2	75.07 (16.69)	82.73 (16.59)	76.59 (20.70)	73.53 (15.81)	3.39 (1)	4.05 (0.936)
	4–5	67.99 (18.83)	75.40 (21.20)	71.82 (25.00)	68.44 (20.07)	3.14 (1.04)	3.87 (1.03)
		*P* = 0.609	*P* = 0.788	*P* = 0.973	*P* = 0.851	*P* = 0.483	*P* = 0.699
Q7^*c*^	1–2	73.76 (17.34)	81.83 (17.59)	75.28 (21.66)	72.38 (16.78)	3.34 (1.01)	3.98 (0.952)
	4–5	66.01 (18.62)	74.31 (20.18)	71.47 (25.41)	67.43 (21.01)	3.16 (1.03)	3.83 (1.04)
		*P* = 0.432	*P* = 0.941	*P* = 0.353	*P* = 0.233	*P* = 0.641	*P* = 0.362
Q8^*d*^	1–2	66.67 (20.82)	76.09 (22.47)	69.08 (25.12)	61.77 (20.96)	3.06 (1.03)	3.65 (1.08)
	4–5	73.90 (16.34)	81.09 (17.08)	77.38 (20.78)	75.67 (16.11)	3.38 (1.03)	4.10 (0.936)
		*P* = 0.263	*P* = 0.801	*P* = 0.849	*P* = 0.173	*P* = 0.208	*P* = 0.701
Q16^*e*^	1–2	74.63 (17.25)	82.51 (18.44)	76.22 (21.84)	73.71 (17.66)	3.41 (1.06)	4.10 (0.951)
	4–5	67.58 (18.32)	75.28 (19.72)	71.65 (23.90)	67.02 (19.47)	3.06 (1.03)	3.78 (1.07)
		*P* = 0.772	*P* = 0.985	*P* = 0.292	*P* = 0.934	*P* = 0.688	*P* = 0.299

Mean (SD).

One Sample T-test for compar means.

One Way ANOVA used for P value.

^a^Are you afraid of a possible war between Romania and the Russian Federation in the near future?

^b^To what extent do you think the Russian Federation planned to invade Romania or another NATO member state?

^c^To what extent do you think Russian President Vladimir Putin will start a nuclear war against NATO member states?

^d^To what extent do you feel protected by the fact that Romania is a NATO member state?

^e^Does the current military conflict in Ukraine have any influence on your life?

1 - To a very small extent; 2 - To a small extent; 4 - To a large extent; 5 - To a very large extent.

## Discussions

As can be seen in the section presenting the results, respondents have different views on the war between Ukraine and the Russian Federation and its potential escalation into a regional, continental or global conflict.

It should be noted that 47% of respondents fear a possible war between the Russian Federation and Romania, while 31.8% believe, to a large and very large extent, that the Russian Federation has planned to invade Romania as well, although there is no information, clues or statements in this regard. Such views are determined by the fact that Romania was under influence of the USSR (15) until 1989, and the Russian Federation wants to regain influence over the former Soviet states (16).

The Russian Federation's nuclear weapons (17), as well as its nuclear policy (18), also create serious anxiety among respondents, with 30.2% believing, to a large and very large extent, that a nuclear attack on Ukraine is possible, while 24.4% considering the scenario of a nuclear attack on NATO member states likely to happen. These fears have also been raised by President Vladimir Putin, who has made a declarative statement about the scenario of a nuclear attack on NATO member states if they were to intervene in support of Ukraine (19). We believe that such a scenario may be possible, but it is unlikely to happen.

The security that NATO provides to Romania (20) is questioned by 23.3% of respondents, who believe that they do not feel protected by the fact that our country is part of this alliance, and 16.7% of respondents consider the presence of NATO troops on the territory of Romania as not beneficial. Such opinions may be based on the fear of a potential war with the Russian Federation as a result of Romania's NATO membership or due to the deployment of some US military capabilities on the Romanian territory. However, such a hypothesis is invalidated, as there are no correlations between variables, by the Kendall and Spearman statistical tests - as shown in Table 12 - performed between the data obtained from the answers to the question aimed at measuring the fear of war (Q4) and the questions measuring the opinion on Romania's NATO membership (Q8) and the presence of NATO troops on the Romanian territory (Q9).

**Table 12:** Correlation of the question that aims to measure the fear of war (Q4) and the questions measuring the opinion on Romania's NATO membership (Q8) and the presence of the Alliance troops on the Romanian territory (Q9).

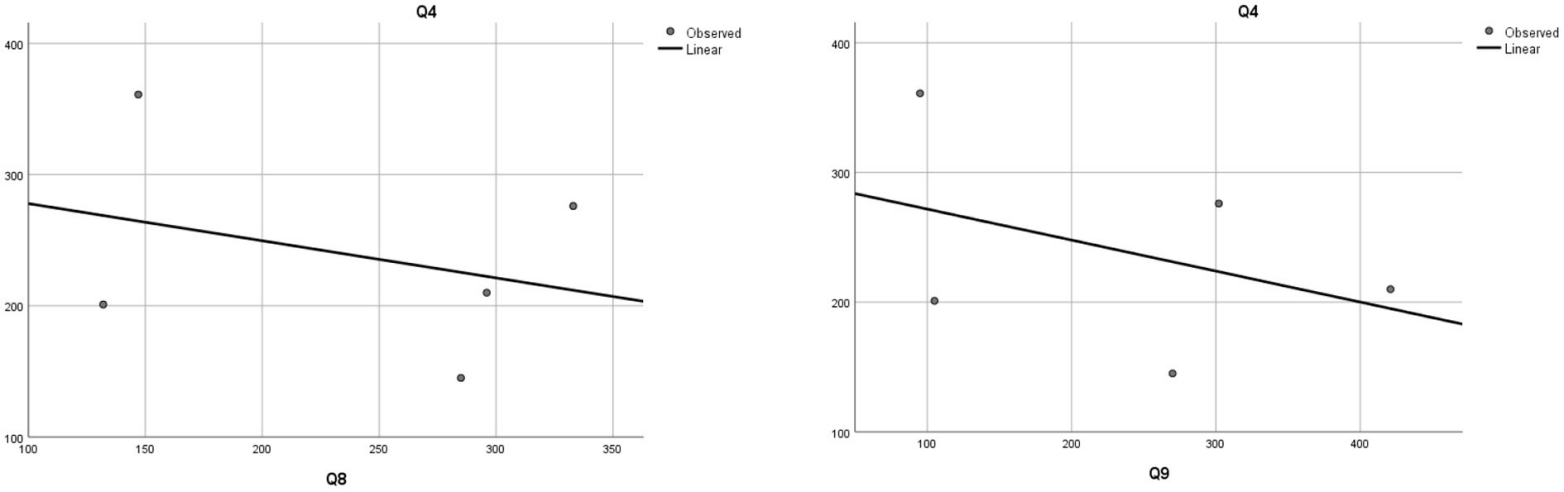					
Q4 - Are you afraid of a possible war between Romania and the Russian Federation in the near future?					
Q8 - To what extent do you feel protected by the fact that Romania is a NATO member state?					
Q9 - To what extent do you consider the presence of NATO military capabilities on the territory of our country to be beneficial for Romania's security?					
Kendell	Correlation coefficient	0.200	Kendell	Correlation coefficient	−0.200
	Sig. (2-tailed)	0.624**		Sig. (2-tailed)	0.624**
Spearman	Corelation coefficient	0.200	Spearman	Correlation coefficient	−0.200
	Sig. (2-tailed)	0.747**		Sig. (2-tailed)	0.747**


^**^Correlation is significant at the 0.01 level (2-tailed).

For a correlation to be very strong, the correlation coefficient must be as close as possible to 1, and sig. as close as possible to 0.

15.8% of participants in the study believe that NATO member states will not intervene in a potential invasion of Romania by the Russian Federation, and 23.1% believe that the US will not intervene in the event of a war against Romania.

In this context, it should be mentioned that Romania's security is at the highest level ever reached, being strengthened by the military capabilities of the allies present on its territory, and with the outbreak of the invasion of Ukraine, NATO leaders decided to set up four battle groups to be deployed in Romania, Hungary, Bulgaria and Slovakia as part of the Alliance's response to Russia's unprovoked invasion of Ukraine (21).

The fear of a potential escalation of the conflict in Ukraine is strongly felt among the participants with 38.3% of them believing, to a large and very large extent, that the war started by the Russian Federation has affected their lives. There is also a strong correlation (Table 13) between those who have a high fear of a war between Romania and the Russian Federation in the near future (Q4) and people who believe that the invasion of the Russian Federation in Ukraine has affected their lives (Q16).

**Table 13:** Correlation of answers with 4 and 5 to the questions concerning the fear of a future war in Romania and the Russian Federation and the opinion of the participants on the influence of the conflict in Ukraine on their lives.

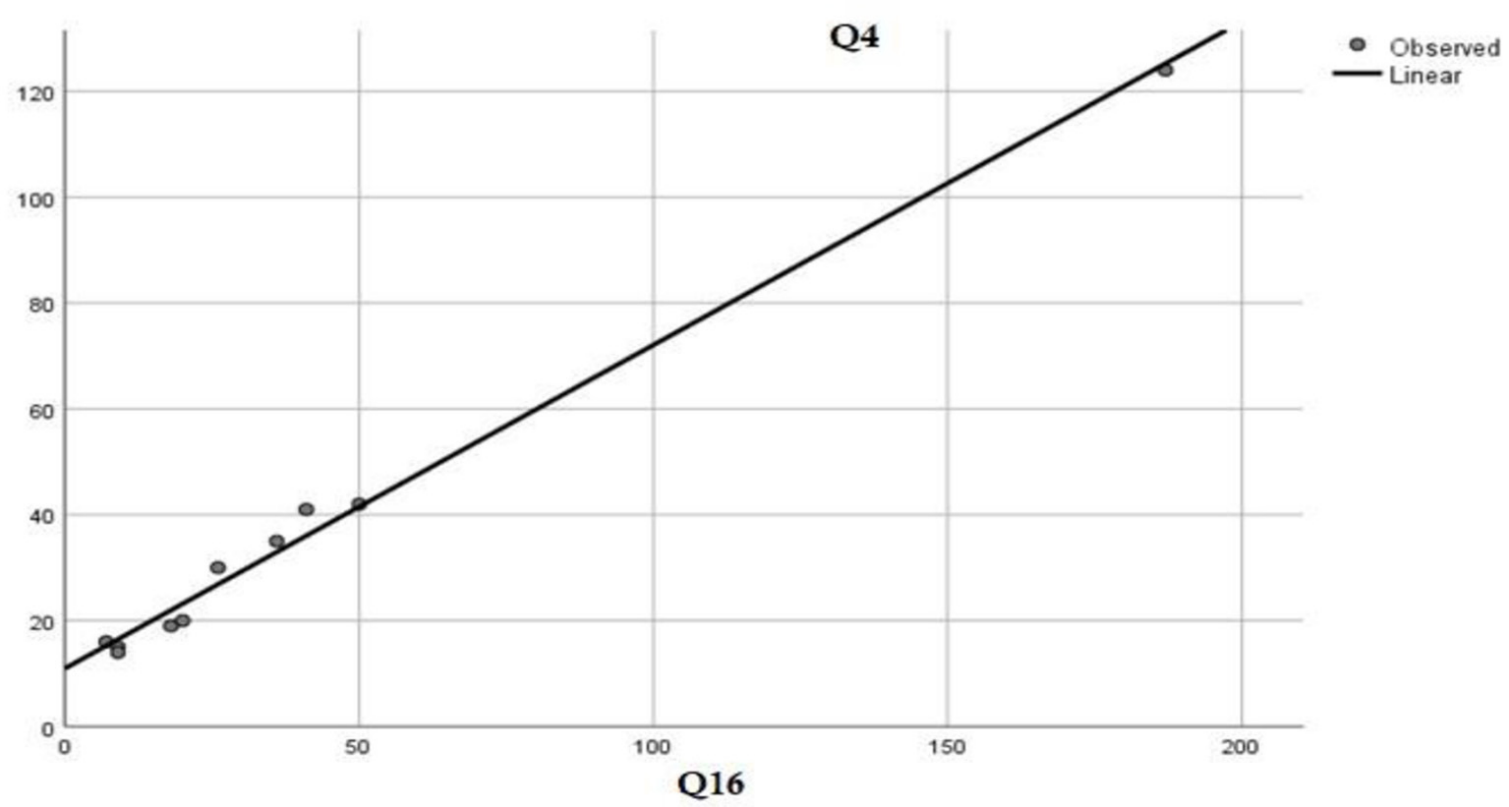		
Q4 - Are you afraid of a possible war between Romania and the Russian Federation in the near future?		
Q16 - Does the current military conflict in Ukraine have any influence on your life?		
Kendell	Correlation coefficient	0.899
	Sig. (2-tailed)	0.000**
Spearman	Correlation coefficient	0.960
	Sig. (2-tailed)	0.000**

^**^ Correlation is significant at the 0.01 level (2-tailed).

For a correlation to be very strong, the correlation coefficient must be as close as possible to 1, and sig. as close as possible to 0.

Based on the results of this study, the highest average satisfaction among the four domains of the WHOQOL-BREF is represented by the “Psychological” domain of the category of people who have the least fear about a potential future war between Romania and the Russian Federation (83.62 ± 17.48). On the contrary, the lowest average is represented by the “Environment” domain for the category of people who do not feel protected by the fact that Romania is a NATO member state (61.77 ± 20.96).

Following the comparative analysis of the medians of the major domains, we found that in all major domains, females have a lower score than that recorded in the case of men, with a significant difference in the “Physical” domain (Male: 75.45 ± 17.47, Female: 69.16 ± 18.10). Studies in the literature suggest clear differences in the approach to stress between women and men, and these differences can have multiple biological, psychological or social explanations. Incidence rates for depression and anxiety are higher in women (22). However, the risk of developing an affective or anxiety disorder in men is high, and the lower incidence of these rates among the male population does not reveal the existence of a stress protection factor or an appropriate coping strategy specific to men, but rather emphasizes the low referral rate of men to psychiatric services. This low referral rate of men to psychiatric services prevents diagnosis and implicitly the beginning of a treatment which leads to a negative evolution and a poor prognosis.

The studies of the participants in this research (secondary studies vs. university studies) do not reveal significant differences. However, participants with higher education had a higher average score in three out of four domains, except for the “Physical” domain, where participants with secondary education scored 71.08 ± 17.96 compared to 70.74 ± 18.13, the score obtained by participants with higher education (23). The level of education is generally a protective factor that allows individuals to identify an accurate picture of the situation and the level of risk that exists. In other words, it is assumed that people with a high level of education have sufficient intellectual potential to develop coping strategies superior to those with no education. However, in the present armed conflict, things are different. The element of surprise has struck everyone, given that the vast majority of the population had not considered the existence of a real war. Many people considered this scenario to be impossible in an era of civilization and the rule of law, democracy and dignity. The outbreak of the armed conflict reset the moral values and mechanisms of psychosocial adaptation of the population, and this sudden, violent and surprising “reset” was accompanied by a wide range of individual interpretations. From everyday medical practice it is observed that the behavioral responses of patients are always proportional to their educational level. This is probably how the insignificant differences in this chapter of our research can be explained.

The rural residence environment shows a higher average score in all four major domains compared to the participants living in urban areas, with the highest score in the “Psychological” domain (80.77 ± 18.93), and the lowest in the “Environment” domain (71.20 ± 19.22). The residence environment is undoubtedly an important socio-demographic indicator in terms of stress management. In Romania, people living in rural areas have limited access to authentic sources of information, and the risk of misinformation is huge. The presentation of false, inaccurate, contradictory news, the so-called “fake-news” has a strong anxiety-provoking effect, significantly altering the quality of life (24).

The evaluation of the data collected shows that there are no significant differences in the indices for assessing the quality of life of respondents across the four major domains, in relation to age groups. However, it should be noted that the group of respondents aged <30 years is characterized by a score of 76.93 (20.29) in the “Psychological” domain, about 4 pp (percentage points) less than the score of 80.71 (17.62) recorded in the category of respondents aged > 30 years. Regarding the “Evironment” domain, the situation is diametrically opposed, the group of respondents aged <30 years is characterized by a score of 72.14 (18.30) being about 4 pp higher than the score of 68.92 (18.49) recorded in the category of respondents aged> 30 years.

The four major domains calculated based on the answers to the specific questions in the first part of the questionnaire (Q4, Q7, Q8 and Q16) reveal, according to Table 11, that the research hypothesis proposed in this study is supported. Thus, participants who show an increased fear of a potential war between Romania and the Russian Federation, a potential nuclear attack on Ukraine or on a NATO member state, or consider that the war in Ukraine has affected their lives, have a significantly lower average, in all four major domains, compared to people who do not show such fears. The maximum average is obtained in the “Psychological” domain (83.62 ± 17.48), for people who have no fears about a future conflict, and the minimum average in the “Physical” domain (67.23 ± 18.33) for people who believe that there will be a conflict between Romania and Russia in the near future. Poikolainen, Kanerva and Lonnqvist consider that no study has examined whether the fear of nuclear war is an expression of intrapsychic factors coming from the deep irrational layers of the unconscious or a response to a real danger (25). Thus, neither can we launch a theory on these issues.

As for Question Q5, it was assigned the role of control for Question Q4, with the aim of having a very clear view of the results of the participants' quality of life. Thus, from a theoretical point of view, we should have obtained similar results for the quality of life of those who answered similarly (1–2 and 4–5) to the two questions. The maximum difference for those who answered 4 and 5 is 0.71 for the “Environment” domain, and for those who answered 1 and 2, the maximum difference is 1.41 for the “Physical” domain.

The largest difference between the average scores for the same major domain is the “Physical” domain in Q4. Participants who have fears about a future conflict between Romania and the Russian Federation (67.23 ± 18.33) have an average of 9.25 times lower than those who do not have such fears (76.48 ± 17.03).

## Research limitations

This study has many positive aspects, but also some limitations, as it is among the few existing studies addressing such an issue.

A limitation of the research is the application of the survey in the online environment. Although our research is qualitative, based on the number of responses obtained, in terms of sample, there is a possibility that biased respondents may self-select. Also, only people who had access to the Internet could answer the questionnaire.

## Conclusions

The results of the study show that the indices of the quality of life of people in Romania, as a state in the vicinity of a military conflict with the potential for escalating, are negatively affected by the fears of people who believe that the war in Ukraine will escalate into a regional or global conflict.

Although there are no significant differences in the assessment indices of the quality of life of respondents in the four major domains in relation to age groups, it should be noted that the group of respondents aged <30 years is more affected by the impact on the “physical” domain of the military conflict in the vicinity of Romania, while the category of respondents aged> 30 is more affected by the alteration of environmental factors, both domains characterizing the basic needs of the person. According to the data obtained, it can be hypothesized that during an armed conflict, citizens in its vicinity of the conflict are more interested in providing basic needs (the “Physical” and “Environment” domains) than for higher needs (the “Psychological” and “Social” domains).

The comparative analysis of the medians of the major domains shows that in all major domains, females score lower than males with a significant difference in the “Physical” domain.

On the other hand, the rural residence environment has a higher average score in all four major domains compared to the urban residence envrionment, with the highest score in the “Psychological” domain, and the lowest in the “Environment” domain.

In relation to the respondent's educational background, there are no significant differences in the assessment indices of the quality of life of respondents in the four domains, with the greatest differences for higher education graduates in the “Psychological” and “Environment” domains.

Finally, according to the data presented in the results section and their interpretations, we believe that the research hypothesis, stated in the introductory section, is valid, the invasion of Ukraine by the Russian Federation influencing the quality of life of adults in Romania due to fears developed by the participants.

## Data availability statement

The datasets presented in this study can be found in online repositories. The names of the repository/repositories and accession number(s) can be found in the article/Supplementary material.

## Ethics statement

The study was conducted according to the guidelines of the Declaration of Helsinki, and approved by the Institutional Review Board (or Ethics Committee) of Constantin Brâncuşi University of Târgu Jiu. Written informed consent for participation was not required for this study in accordance with the national legislation and the institutional requirements.

## Author contributions

All authors listed have made a substantial, direct, and intellectual contribution to the work and approved it for publication.

## Conflict of interest

The authors declare that the research was conducted in the absence of any commercial or financial relationships that could be construed as a potential conflict of interest.

## Publisher's note

All claims expressed in this article are solely those of the authors and do not necessarily represent those of their affiliated organizations, or those of the publisher, the editors and the reviewers. Any product that may be evaluated in this article, or claim that may be made by its manufacturer, is not guaranteed or endorsed by the publisher.
